# ﻿*Phalaenopsiszhanhouana* (Orchidaceae, Vandeae), a new species from Yunnan, China

**DOI:** 10.3897/phytokeys.237.112270

**Published:** 2024-01-22

**Authors:** Shiyu Qin, Hanchen Wang, Yajun Wang, Chongbo Ma, Zan Li, Boyun Yang, Xiaohua Jin

**Affiliations:** 1 School of Life Sciences, Nanchang University, Xuefudadao 999, Shajing, Nanchang, Jiangxi, 330031, China Institute of Botany, Chinese Academy of Sciences Beijing China; 2 State Key Laboratory of Plant Diversity and Prominent Crops, Institute of Botany, Chinese Academy of Sciences, Nanxincun 20, Xiangshan, Beijing, 100093, China Nanchang University Nanchang China; 3 China National Botanical Garden, Beijing, 100093, China China National Botanical Garden Beijing China; 4 University of Chinese Academy of Sciences, Beijing, 100093, China University of Chinese Academy of Sciences Beijing China

**Keywords:** China, new species, *Phalaenopsiszhanhouana*, Xichou County, Yunnan

## Abstract

A new species of Orchidaceae, *Phalaenopsiszhanhouana*, from Xichou County, Yunnan, China, is described and illustrated. The novelty is close to *P.taenialis*, *P.wilsonii*, and *P.stobartiana*, but differs from them by having a distinct, fleshy anterior callus with a deeply lobed apex at the base of the labellum and lateral lobes of labellum reflexed and facing outward.

## ﻿Introduction

The moth orchid genus, *Phalaenopsis* Blume, comprises approximately 80 recognized species (https://powo.science.kew.org/) and is extraordinarily prominent in the field of horticulture. *Phalaenopsis* is distributed in India, Southeast to East Asia, and Australia, with most of the diversity in Indonesia and the Philippines (Pridgeon et al. 2014). Recent molecular results based on ITS nrDNA and plastid regions (*trnL* intron, *trnL-F* spacer, and *atpB*-*rbcL* spacer) indicated that the number of pollinia was not a good morphological character to distinguish *Phalaenopsis* from its alliance, such as *Doritis* Lindl., *Kingidium* P.F.Hunt., and *Nothodoritis* Z.H. Tsi, and proposed broadening *Phalaenopsis* to include its alliance (Christenson 2001; Padolina et al. 2005; Tsai et al. 2005; Yukawa et al. 2005; Tsai et al. 2010; Pridgeon et al. 2014). *Phalaenopsiss.l.* is characterized by roots more or less depressed and verrucose, stem short, leaves usually elliptic and fleshy, lip three lobed, column usually with column foot, pollinia two or four.

Based on molecular data and morphological characters, such as the presence or absence of column foot and the number of pollinia, *Phalaenopsis* was subdivided into four subgenera, subgen. Parishianae (Sweet) Christenson (26 spp.), subgen. Phalaenopsis Blume (45 spp.), subgen. Hygrochilus (Pfitzer) Kocyan & Schuiteman (5 spp.) and subgen. Ornithochilus (Lindl.) Kocyan & Schuiteman (4 spp.) (Kocyan and Schuiteman 2014; Pridgeon et al. 2014; Higgins and Alrich 2015).

The subgenus Parishianae is mainly distributed in India, Southeast to East Asia (Pridgeon et al. 2014), with species morphologically characterized by small plant size, few-flowered inflorescence, small scarious floral bracts, and biseriate callus (Pridgeon et al. 2014). There are 25 *Phalaenopsis* species in four subgenera in China (Zhou et al. 2021; Ma et al. 2022), of which 14 species belong to the subgenus Parishianae (Chen and Wood 2009; Zhou et al. 2021). During our fieldwork in Yunnan Province, China, in April 2023, a new species of Phalaenopsis belonging to subgenus Parishianae (Sweet) Christenson was found in evergreen broad-leaved forests and is described here.

## ﻿Materials and methods

Morphological characters of the new species were observed, measured with a ruler (precision: 1 mm), and photographed based on living plants. Molecular phylogenetic analyses were conducted using one nuclear (nrITS) and four plastid markers (*matK*, *trnL*, *trnL-F*, and *atpB-rbcL*). Genomic DNA was extracted from the newly collected specimen of *Phalaenopsis* (silica dried) using the modified cetyltrimethylammonium bromide (CTAB) method (Li et al. 2013). Sequencing library was generated using Rapid Plus DNA Lib Prep Kit for Illumina and then delivered to Novogene Company (Beijing, China) for 150 bp paired-end sequencing on the Illumina HiSeq 2500 platform. Approximately 5 Gb of raw sequencing data were generated for the collected specimen. Plastid genome and ITS were assembled using GetOrganelle v.1.7.1 with Illumina sequencing reads as input and under default parameters (Jin et al. 2020), respectively. The assembled plastid genome was annotated using Geneious Prime v.2023.0.4 (https://www.geneious.com) and manually checked with *P.lobbii* (NC_059699) and *P.stobartiana* (NC_059917) as references. Four plastid markers (*matK*, *trnL*, *trnL-F*, and *atpB-rbcL*) were extracted from plastid genome using Geneious Prime v.2023.0.4.

Sixty-two species of *Phalaenopsis* were used for phylogenetic analyses. Two species, *Cleisostomawilliamsonii* (Rchb. f.) Garay and *Pelatantheriarivesii* (Guillaumin)Tang & F. T.Wang, were used as outgroup based on previous results (Chase et al. 2015; Li et al. 2019; Ma et al. 2022). In total, 225 sequences from 64 Orchidaceae species were downloaded from NCBI (Suppl. material 1: table S1). The combined matrix thus includes 229 sequences for the five markers, belonging to 65 species. Sequence alignment, supermatrix generation, and substitution model selection were performed using PhyloSuite (Zhang et al. 2020). GTR+F+I+G4 was selected as the best model for *matK*, *atpB-rbcL*, *trnL-F*, and *trnL*, and GTR+F+G4 for ITS, respectively. Bayesian Inference of phylogeny was performed using MrBayes v.3.2.7a on XSEDE in the CIPRES Science Gateway online web server (Miller et al. 2010). Two separate Markov Chain Monte Carlo (MCMC) analyses were performed 1,000,000 generations and sampling every 1000 generations. Maximum likelihood (ML) analyses were performed locally using IQTree2 (Minh et al. 2020). Support values for the clade were estimated using 1,000,000 bootstrap replicates.

## ﻿Results

**Figure 1.**
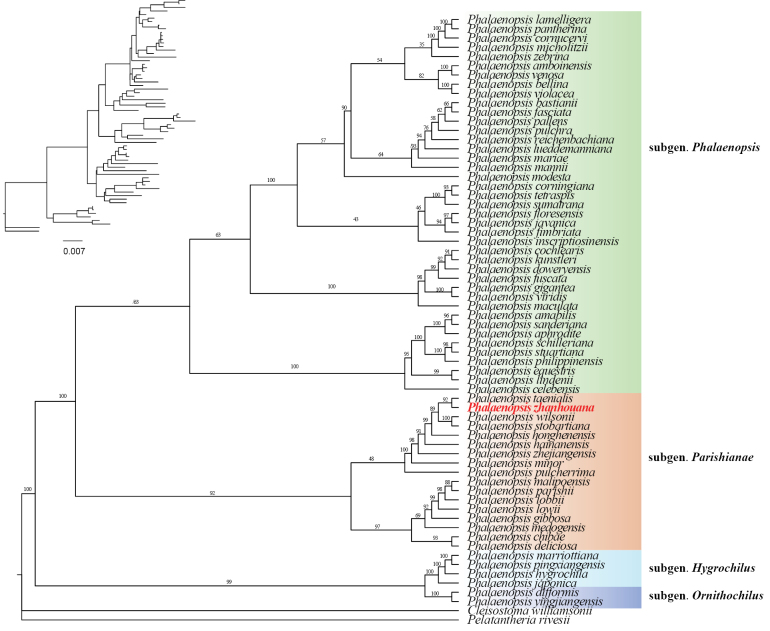
Phylogram of Maximum Likelihood based on nrITS and plastid DNA markers (*matK*, *trnL*, *trnL-F* and *atpB-rbcL*). Numbers above branches indicate bootstrap percentages (BS) for ML.

Phylogram of Maximum Likelihood based on nrITS and plastid DNA markers were used to illustrate the phylogenetic position of the new species. Phalaenopsis sp. nov. is nested within subgen. Parishianae and sister to *P.taenialis* with high support (PP = 1, BSML = 92; Fig. 1, Suppl. material 2: fig. S1). *Phalaenopsis* sp. nov. and *P.taenialis* together formed a clade sister to the clade consisting of *P.stobartiana* and *P.wilsonii* with high support (PP = 0.994, BSML = 89; Fig. 1, Suppl. material 2: fig. S1).

The new species is morphologically close to *P.taenialis*, *P.wilsonii*, *and P.stobartiana* by sharing lip with two seriate of calli at base, lip more or less with spur, lateral lobes more or less erect. *Phalaenopsis* sp.nov., however, differs from its relatives by having a bifurcated, fleshy, yellow anterior callus, and lateral lobes flipping outward and center with large calli.

### ﻿Key to *Phalaenopsiszhanhouana* sp. nov. and its relatives

**Table:** 

1a	Spur not prominent, apparently absent or forming a small nipple-shaped structure; middle lobe more or less convex	**2a**
2a	Lateral lobes flipping backward and centre with large yellow calli; anterior callus yellow, bifurcate	**1. *P.zhanhouana* sp.nov.**
2b	Lateral lobes without large calli; anterior callus purple, bifid; lobelets linear	**3a**
3a	Lip mid-lobe obcordate with a central apical fleshy knob	**2. *P.wilsonii***
3b	Lip mid-lobe not obcordate, without a terminal notch	**4a**
4a	Petals and sepals deep green, lip purple; mid-lobe without any conspicuous constriction	**3. *P.stobartiana***
4b	Flowers rose-pink; mid-lobe with a conspicuous constriction	**5a**
5a	Lip mid-lobe flared below apex producing a 3-lobulate mid-lobe	**4. *P.hainanensis***
5b	Lip mid-lobe widest below apex, apical margin reflexed along mid-vein, forming a subtubular apex that may appear emarginate in natural position	**5. *P.honghenensis***
1b	Spur prominent, a continuation of angle formed by junction of lip mid-lobe and lateral lobes; lip midlobe flat	**6. *P.taenialis***

### ﻿Taxonomy

#### ﻿ Phalaenopsis zhanhouana

Taxon classificationPlantaeAsparagalesOrchidaceae

X.H.Jin & S.Y.Qin sp. nov.

AC641B8A-C2F1-5D36-991E-A37B11195946

urn:lsid:ipni.org:names:77334958-1

Figs 2 , 3 吉氏蝴蝶兰

##### Type.

China. Yunnan, Wenshan Ctiy, Xichou County, alt. 1496 m, 11 Apr 2023, *Xiaohua Jin & Shiyu Qin 40050* (holotype, PE!).

##### Diagnosis.

*Phalaenopsiszhanhouana* is similar to *P.wilsonii*, but differs from it by having a bifurcated yellow, fleshy anterior callus, lateral lobes with large calli and flipping outward (Table 1).

**Table 1.** Morphological comparison of *Phalaenopsiszhanhouana* and close taxa.

	*P.zhanhouana*	*P.wilsonii*	*P.stobartiana*	*P.taenialis*
Flower color	white with pale pink ribs	white with pale pink ribs or complete pale pink.	**sepals and petals apple-green to dark olive-green.**	petals pale pink, lip and anther cap rose-purple.
Leaves	**no leaves at anthesis.**	leaves often deciduous in dry season.	leaves often deciduous during dry season, but present at anthesis.	leaves often deciduous at anthesis or during dry season
Lateral sepals	lateral sepals elliptic, acute at apex, obtuse	lateral sepals obovate-elliptic, similar and equal to middle sepal.	lateral sepals slightly oblique, ovate-elliptic, subacute.	lateral sepals subelliptic, **base adnate to column foot**, apex obtuse.
Lateral lobes of lip	lateral lobes **flipping outward**, adaxial center with a big callus	lateral lobes erect, adaxially **with an incised-tipped keel**.	lateral lobes erect, narrow, slightly constricted at middle	lateral lobes **adaxially with a slightly thickened longitudinal ridge close to proximal margin**
callus	**yellow, fleshy, bifurcated.**	purple; anterior callus deeply lobed at apex; lobelets linear and long	purple; concave adaxially and distinctly convex abaxially on disk.	purple; ligulate, deeply bifid; lobelets linear and long, attached to front wall at base of mid-lobe.

##### Description.

Epiphytic plants. Roots fleshy, developing from the base or lower parts of the stem, elongated, flattened, densely verrucose and prostrate along trunks. Stem very short, covered by tubular sheath at base. Leaves unseen. Inflorescence developing from the base of stem, suberect or arching, ca. 4.5 cm long, unbranched, with 3 laxly arranged flowers. Floral bracts ovate-triangular, 4–5 mm long. Flowers white with pale pink rib or white, 3–4 cm in diameter. Dorsal sepal broadly elliptic or spoon-shaped, ca. 2 × 1 cm, with semi-transparent veins abaxially; lateral sepals elliptic, acute at apex, slightly curved toward labellum, lilac spots at the apex in the dorsal, ca.1.8–2.0 × 0.9–1.1 cm, obtuse and notched at base. Petals spathulate, ca. 1.8–2.0 × 0.9–1.1cm, apex obtuse. Labellum three-lobed, clawed at the base, ca. 1–2 mm long; lateral lobes of labellum erect, purple, 0.5 cm long, flipping outward, adaxially center with a big callus; mid-lobe of labellum obcordate, ca. 1.3–1.5 × 0.9–1.1 cm, deep purple, with white stripes at the center, base with a yellow fleshy protuberant anterior callus; anterior callus deeply lobed at apex. Column subparallel to midlobe of labellum, lavender, ca. 0.6 cm long, with triangular wings; pollinarium yellow.

**Figure 2.**
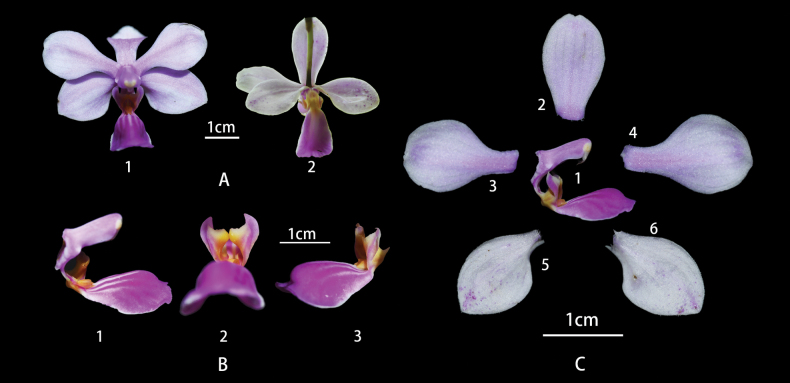
Flowers of *Phalaenopsiszhanhouana* X.H.Jin & S.Y.Qin, sp. nov. **A** front view of flower (1) rear view of flower (2) **B** column and lip; lateral view of column and lip, appendage and lateral lobes (1), front view of lateral lobes (2), lateral view of lateral lobes and mid-lobe (3) **C** petal, sepal and lip, lip (1), dorsal sepal (2), petal (3.4), lateral sepals (5.6). Photographed by Xiaohua Jin.

##### Etymology.

The epithet *zhanhouana* was designated in honor of the Chinese botanist Zhanhuo Tsi.

##### Distribution and habitat.

*Phalaenopsiszhanhouana* is currently known only from the type locality in Xichou, Yunnan, China. It is epiphytic on trunks and twigs at elevations 1400–1500 m in evergreen broad-leaved forests.

**Figure 3.**
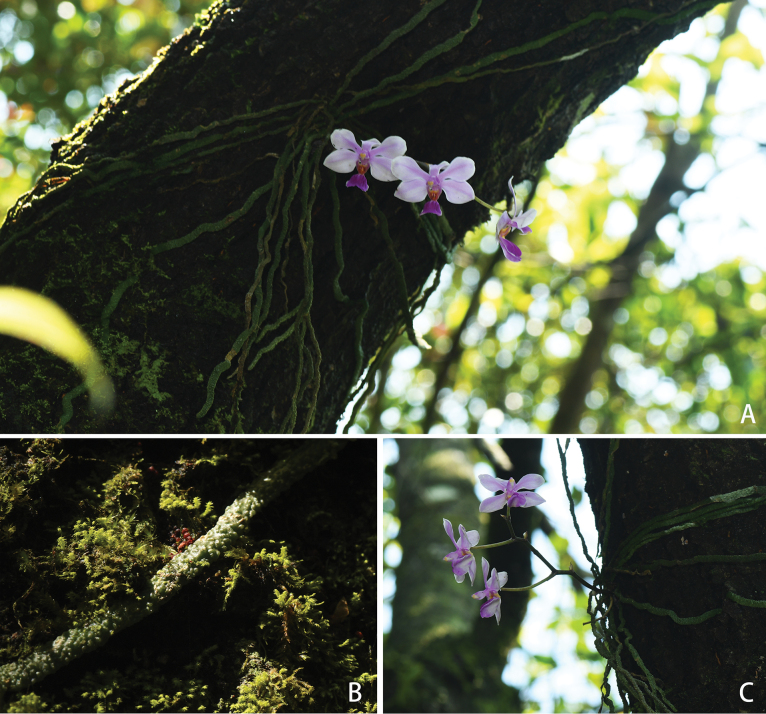
Habitat and plants of *Phalaenopsiszhanhouana* X.H.Jin & S.Y.Qin, sp. nov. **A** front view **B** roots **C** lateral view. Photographed by Xiaohua Jin.

##### Phenology.

Flowering in March and April.

##### Conservation status.

*Phalaenopsiszhanhouana* grows in evergreen broad-leaf forests in Xichou County Yunnan Province, China. One subpopulation of about 10 individuals was discovered during our fieldwork. The habitat has been severely fragmented due to the development of agriculture. During our survey in nearby forests, we did not find any additional subpopulation of the new species. According to IUCN criteria v15.1 (IUCN 2022), we putatively assessed this new species as Critically Endangered CR C2a(i).

## Supplementary Material

XML Treatment for Phalaenopsis zhanhouana

## ﻿Additional information

### Author ORCIDs

Shiyu Qin https://orcid.org/0009-0008-2680-6789

Hanchen Wang https://orcid.org/0000-0002-4690-8052

Yajun Wang https://orcid.org/0009-0001-1035-3964

Chongbo Ma https://orcid.org/0000-0001-5869-8649

Zan Li https://orcid.org/0009-0000-3098-1418

Boyun Yang https://orcid.org/0000-0003-2123-0027

Xiaohua Jin https://orcid.org/0000-0002-9987-5602

### Data availability

All of the data that support the findings of this study are available in the main text or Supplementary Information.

## Supplementary materials

Supplementary material 1 GenBank accession numbers for sequences used for phylogenetic analyses Data type docx https://binary.pensoft.net/file/973903This dataset is made available under the Open Database License (http://opendatacommons.org/licenses/odbl/1.0/). The Open Database License (ODbL) is a license agreement intended to allow users to freely share, modify, and use this Dataset while maintaining this same freedom for others, provided that the original source and author(s) are credited.Shiyu Qin, Hanchen Wang, Yajun Wang, Chongbo Ma, Zan Li, Boyun Yang, Xiaohua Jin

Supplementary material 2 Phylogram of Bayesian Inference (BI) based on nrDNA ITS and plastid sequences (*matK*, *trnL*, *trnL-F*, and *atpB-rbcL*) Data type pdf Explanation note Numbers above branches indicate posterior probabilities (PP) for BI. https://binary.pensoft.net/file/973904This dataset is made available under the Open Database License (http://opendatacommons.org/licenses/odbl/1.0/). The Open Database License (ODbL) is a license agreement intended to allow users to freely share, modify, and use this Dataset while maintaining this same freedom for others, provided that the original source and author(s) are credited.Shiyu Qin, Hanchen Wang, Yajun Wang, Chongbo Ma, Zan Li, Boyun Yang, Xiaohua Jin
