# Supplementary material for: ﻿Phalaenopsiszhanhouana (Orchidaceae, Vandeae), a new species from Yunnan, China
Source: PhytoKeys. 2024 Jan 22;237:153–60. doi: 10.3897/phytokeys.237.112270 (PMC10825969; doi:10.3897/phytokeys.237.112270)
Supplement: Supplementary material 1 — GenBank accession numbers for sequences used for phylogenetic analyses [file phytokeys-237-153_article-112270__-s001.docx]

|  |  | ***ITS*** | ***matK*** | ***trnL* intron** | ***trnL-F* spacer** | ***atpB-rbcL*** |
| --- | --- | --- | --- | --- | --- | --- |
| 1 | *Phalaenopsis amabilis* | AY391535 | EU256323 | AY265742 | AF533472 | AY389440 |
| 2 | *Phalaenopsis amboinensis* | AF537006 | AY498548 | AY265743 | AF533458 | AY389422 |
| 3 | *Phalaenopsis aphrodite* | AY391550 | EU256324 | AY265744 | AF533473 | AY389441 |
| 4 | *Phalaenopsis appendiculata* | AY912218 | AY526086 | DQ194981 | DQ194996 | AY389421 |
| 5 | *Phalaenopsis bastianii* | AY912219 | MT518644 | AY265745 | AF533452 | AY389416 |
| 6 | *Phalaenopsis bellina* | AY390238 | EU251961 | AY265746 | AF533467 | AY389433 |
| 7 | *Phalaenopsis braceana* | KJ733432 | KJ733589 | KJ733669 | AY266119 | AY389405 |
| 8 | *Phalaenopsis celebensis* | AF537014 |  | AY265799 | AF533466 | AY389432 |
| 9 | *Phalaenopsis chibae* | AB217572 | AB217748 | ay265800 | AF533447 | AY389412 |
| 10 | *Phalaenopsis cochlearis* | AY912221 | MT518645 | AY265749 | DQ194997 | AY389400 |
| 11 | *Phalaenopsis corningiana* | AY390246 |  | AY265750 | AF533448 | AY389413 |
| 12 | *Phalaenopsis cornu-cervi* | AY912222 | KC823038 | AY265751 | AF533445 | AY389408 |
| 13 | *Phalaenopsis deliciosa* | DQ091670 | KJ733590 | KJ733670 | AF533468 | AY389434 |
| 14 | *Phalaenopsis doweryensis* | AY912224 |  | AY265753 | AF533485 | AY389395 |
| 15 | *Phalaenopsis equestris* | AY912225 | AF263677 | AY265754 | AF533464 | AY389430 |
| 16 | *Phalaenopsis fasciata* | AB217574 | AB217750 | AY265755 | AF533490 | AY389401 |
| 17 | *Phalaenopsis fimbriata* | AF537013 |  | AY265756 | AF533465 | AY389431 |
| 18 | *Phalaenopsis floresensis* | AY912227 | MT518651 | AY265797 | AF533462 | AY389428 |
| 19 | *Phalaenopsis fuscata* | AY228498 | MT518652 | AY265757 | AF533478 | AY389388 |
| 20 | *Phalaenopsis gibbosa* | AY912228 |  | AY265758 | AF533461 | AY389427 |
| 21 | *Phalaenopsis gigantea* | AF537031 | MT518653 | AY265759 | AF533484 | AY389394 |
| 22 | *Phalaenopsis hainanensis* | DQ091671 |  | KP976028 | KP976036 | KP976017 |
| 23 | *Phalaenopsis honghenensis* | AY912229 |  | DQ195040 | DQ194995 | DQ195013 |
| 24 | *Phalaenopsis hygrochila* |  | MN124430 | MN124430 | MN124430 | MN124430 |
| 25 | *Phalaenopsis inscriptiosinensis* | AY912230 | MT518656 | AY265761 | AF533459 |  |
| 26 | *Phalaenopsis japonica* | AB217585 | MG490267 | KP976023 | KP976031 | KP976012 |
| 27 | *Phalaenopsis javanica* | AY912231 |  | AY265763 | AF533455 | AY389424 |
| 28 | *Phalaenopsis kunstleri* | AY912232 |  | AY265764 | AF533486 | AY389396 |
| 29 | *Phalaenopsis lamelligera* | AY912233 | EU179845 | AY265765 | AF533477 |  |
| 30 | *Phalaenopsis lindenii* | AY912234 |  | AY265766 | AF533480 | AY389390 |
| 31 | *Phalaenopsis lobbii* | DQ091673 | NC_059699 | AY265767 | AF533474 | AY389442 |
| 32 | *Phalaenopsis lowii* | KJ733434 | KJ733591 | AY265795 | AY266117 | AY389439 |
| 33 | *Phalaenopsis lueddemanniana* | AY912237 |  | AY265768 | KF558184 | AY389436 |
| 34 | *Phalaenopsis maculata* | AF537008 | MT518657 | AY265798 | AF533460 | AY389426 |
| 35 | *Phalaenopsis malipoensis* | KP976021 | NC_060875 | KP976029 | KP976037 | KP976018 |
| 36 | *Phalaenopsis mannii* | KJ733435 | AY121744 | KJ733672 | AF533469 | AY389435 |
| 37 | *Phalaenopsis mariae* | AY912239 | MT518659 | AY265770 | AF533449 | AY389414 |
| 38 | *Phalaenopsis marriottiana* | KX579760 | KY966932 | KX579766 | KX579766 |  |
| 39 | *Phalaenopsis micholitzii* | AY912240 | MT518660 | AY265771 | AF533471 | AY389438 |
| 40 | *Phalaenopsis minor* | AY228494 |  | AY265772 | AY266118 | AY389407 |
| 41 | *Phalaenopsis modesta* | AY912242 |  | AY265793 | AF533488 | AY389398 |
| 42 | *Phalaenopsis pallens* | AY912243 |  | AY265773 | AF533479 | AY389389 |
| 43 | *Phalaenopsis pantherina* | AY912244 |  | AY265775 | AF533463 | AY389429 |
| 44 | *Phalaenopsis parishii* | AF537037 |  | AY265774 | AF533491 | AY389402 |
| 45 | *Phalaenopsis philippinensis* | AY912246 |  | AY265776 | AF533446 | AY389411 |
| 46 | *Phalaenopsis pingxiangensis* | KP938431 | KX579763 | KP938432 | KP938433 |  |
| 47 | *Phalaenopsis pulcherrima* | AY912247 | MT518661 | KJ733673 | AF533495 | AY389404 |
| 48 | *Phalaenopsis pulchra* | AY912248 |  | AY265778 | AF533494 | AY389399 |
| 49 | *Phalaenopsis reichenbachiana* | AY912249 | MT518662 | AY265779 | AY266120 | AY389410 |
| 50 | *Phalaenopsis sanderiana* | AY391553 | MT518663 | AY265780 | AF533453 | AY389417 |
| 51 | *Phalaenopsis schilleriana* | AY912250 |  | AY265781 | AF533443 | AY389425 |
| 52 | *Phalaenopsis stuartiana* | AY912251 |  | AY265782 | AF533492 | AY389403 |
| 53 | *Phalaenopsis stobartiana* |  | NC_059917 | NC_059917 | NC_059917 | NC_05997 |
| 54 | *Phalaenopsis sumatrana* | AY390244 | MT518664 | AY265783 | AF533450 | FJ460412 |
| 55 | *Phalaenopsis tetraspis* | AY912252 | MT518665 | AY265784 | AF533456 | AY389419 |
| 56 | *Phalaenopsis venosa* | AY912253 |  | AY265785 | AF533444 | AY389406 |
| 57 | *Phalaenopsis violacea* | AF537033 | MT518666 | AY265796 | AF533487 | AY389397 |
| 58 | *Phalaenopsis viridis* | AY228497 |  | AY265786 | AF533457 | AY389420 |
| 59 | *Phalaenopsis wilsonii* | DQ091672 | AB217751 | AY265787 | AF533475 | OP723311 |
| 60 | *Phalaenopsis zebrina* | AY390252 |  | AY265788 | AY266121 | AY389409 |
| 61 | *Phalaenopsis zhejiangensis* | KJ733437 | KJ33594 | KJ733674 | KJ733674 |  |
| 62 | *Phalaenopsis medogensis* | ON677963 | ON691648 | ON691649 | ON691649 |  |
| 63 | *Cleisostoma williamsonii* | KJ733409 | MN124426 | MN124426 | MN124426 | MN124426 |
| 64 | *Pelatantheria rivesii* | KJ733429 | MN124412 | MN124412 | MN124412 | MN124412 |
| 65 | *Phalaenopsis zhanhouana* | OR461475 | OR544800 | OR544802 | OR544803 | OR544801 |
