# Supplementary figures and images for: ﻿Phalaenopsiszhanhouana (Orchidaceae, Vandeae), a new species from Yunnan, China
Source: PhytoKeys. 2024 Jan 22;237:153–60. doi: 10.3897/phytokeys.237.112270 (PMC10825969; doi:10.3897/phytokeys.237.112270)

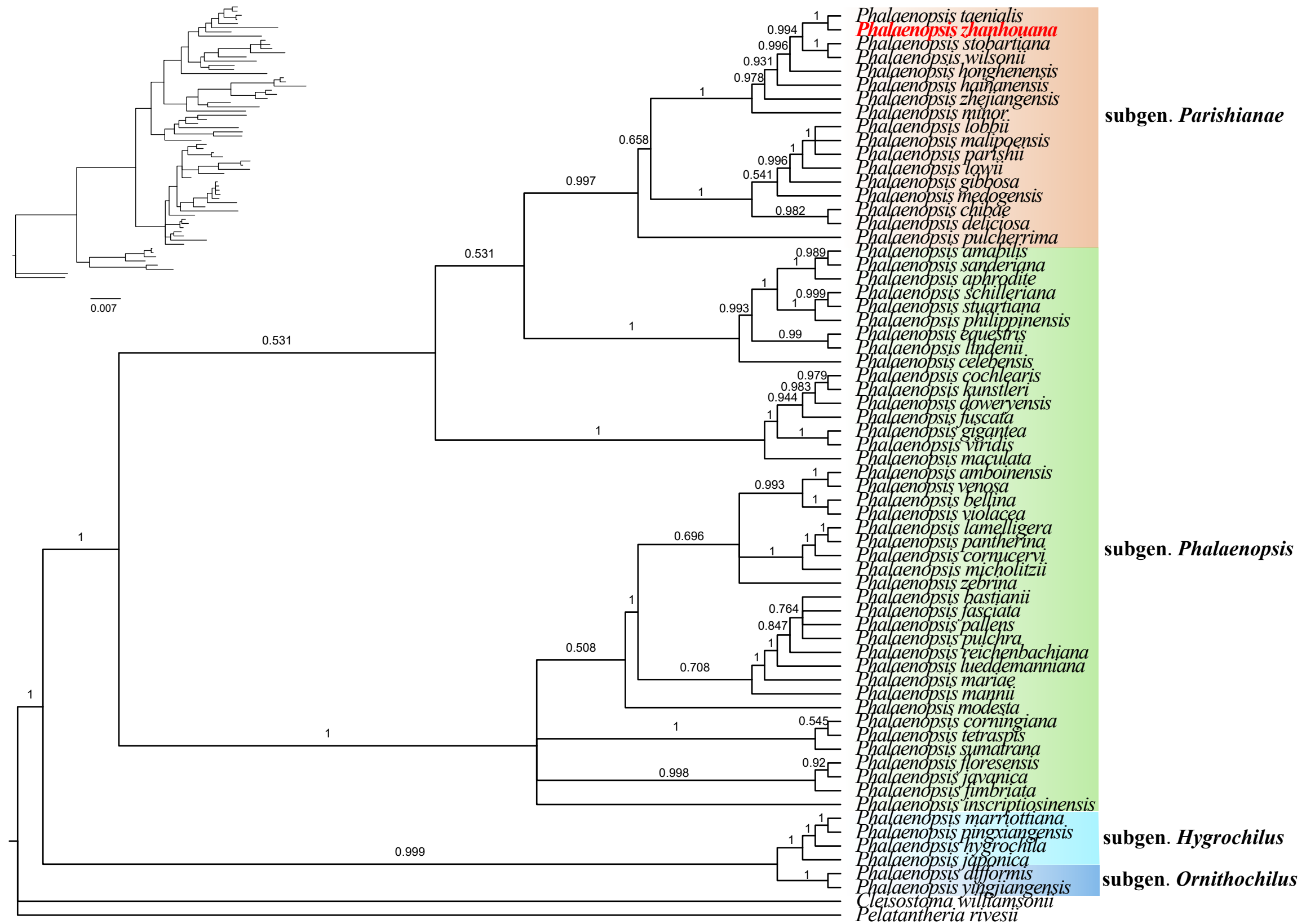

Supplement: Supplementary material 2 — Phylogram of Bayesian Inference (BI) based on nrDNA ITS and plastid sequences (matK, trnL, trnL-F, and atpB-rbcL) [file phytokeys-237-153_article-112270__-s002.pdf]
